# Transfer of inoculum of *Metarhizium anisopliae* between adult *Glossina morsitans morsitans* and effects of fungal infection on blood feeding and mating behaviors

**DOI:** 10.1007/s10340-012-0473-7

**Published:** 2012-12-04

**Authors:** Nguya K. Maniania, Matilda A. Okech, James O. Adino, Jacob O. Opere, Sunday Ekesi

**Affiliations:** The International Centre of Insect Physiology and Ecology (icipe), P.O. Box 30772-00100, Nairobi, Kenya

**Keywords:** *Glossina morsitans morsitans*, *Metarhizium anisopliae*, Entomopathogenic fungus, Tsetse, Mating behavior, Infection, Blood meal, Transfer of inoculum, Reproduction

## Abstract

The transfer of conidia of *Metarhizium anisopliae* between tsetse flies *Glossina morsitans* and the effects of fungal inoculation on mating and blood meal feeding behaviors were investigated in the laboratory. Male or female flies were inoculated with fungal conidia (“donors“) and allowed to pair with fungus-free mate of opposite sex (“recipients”) at 1-day-interval up to three mates. Fungus-treated male or female “donor” flies as well as their mates “recipients” died from fungal infection. However, mortality in male “recipient” flies declined with successive mating, from 82.5 to 32.5 %. Fungus-treated males readily located female flies and mating was successful in most cases comparable to the controls. There were no significant differences (*P* > 0.05) in mean duration of mating, number of jerking movements between fungus-treated and fungus-free males for all the mating lines, except in the number of jerking movements when male flies mated with the 3rd line female flies. Fungus-treated and fungus-free female flies previously mated with treated and non-treated males showed refractoriness during subsequent pairings. The number of fertile female flies was higher (*P* < 0.05) in fungus-free than in fungus-treated treatments, thus producing more pupae. High concentration of fungus (3.0 × 10^6^ conidia ml^−1^) significantly (*P* < 0.05) reduced blood meal intake of flies. This study has shown that fungal infection does not affect the mating behavior of tsetse flies and fly-to-fly contamination does occur during matings. These are important attributes if entomopathogenic fungi have to be used in auto-dissemination strategy and be integrated into sterile insect technique.

## Introduction

The potential of an entomopathogenic fungus to suppress field tsetse populations was recently demonstrated in Lake Victoria, Kenya, against *Glossina fuscipes fuscipes* Newstead (Diptera: Glossinidae) (Maniania et al. 2006). Conidia of *Metarhizium anisopliae* (Metsch.) Sorok. (Hypocreales: Clavicipitaceae) applied in Maniania’s contamination device (Maniania 2002) reduced the fly population by 82.4 % relative to untreated control while “trap and kill” treatment reduced the fly population by 95.8 % during the same experimental period. Five months after removal of the treatments, the number of flies increased considerably in the “trap and kill” treatment compared to the fungus treatment. Fungal infection was observed in fly populations 3 months after the end of the treatment (Maniania et al. 2006). It is hypothesized that horizontal transmission of the inoculum between flies might have contributed to the persistence of the inoculum in the wild population. The transfer of inoculum between fungus-treated and healthy insects has been reported in many Diptera in laboratory assays (Kaaya and Okech 1990; Maniania 1994; Meadow et al. 2000; Scholte et al. 2004; Toledo et al. 2007; Quesada-Moraga et al. 2008). Fungal infection may give rise to behavioral changes such as sexual activity (Watson and Petersen 1993; Schaechter 2000; Dimbi et al. 2009) of the host, which in turn may affect transmission of the pathogen.

Moreover, infection by fungal pathogen has been reported to reduce blood feeding in hematophagous insects (Blanford et al. 2005; Scholte et al. 2006; Ondiaka et al. 2008; Howard et al. 2010; Mnyone et al. 2011) as well as reproductive potential (Eilenberg 1987; Watson and Petersen 1993; Castillo et al. 2000; Meadow et al. 2000; Scholte et al. 2006; Dimbi et al. 2009). The objective of this study was therefore to investigate whether fly-to-fly contamination can take place during mating, and to determine the effects of fungal infection on blood feeding, mating activity, and reproductive potential of *G. m. morsitans* under laboratory conditions.

## Materials and methods

### Insects

*Glossina morsitans morsitans* used in this study were obtained from the Animal Rearing and Quarantine Unit (ARQU) at the International Centre of Insect Physiology and Ecology (*icipe*) and was in its fourth generation. The original stock originated from the International Atomic Energy Agency (IAEA), Vienna, Austria. Flies were maintained in a controlled room temperature (26 ± 2 °C and 70 ± 10 % RH) and fed thrice weekly on rabbits.

### Fungus

The *M. anisopliae* isolate ICIPE 30 used in the study was isolated in 1989 from *Busseola fusca* Fuller (Lepidoptera: Noctuidae) in western Kenya. The virulence of the fungus against *G. m. morsitans* was reported earlier (Maniania and Odulaja 1998). The fungus was maintained on Sabouraud dextrose agar (SDA) plates at room temperature (25 ± 4 °C). Conidia were harvested from the surface culture by scraping. Viability tests were carried out using the technique described by Goettel and Inglis (1997). Conidial suspension (0.1 ml) titrated to 3 × 10^6^ conidia ml^−1^ was spread-plated on 9-cm Petri dishes containing SDA medium. Percent germination was determined by counting the number of germinated conidia/100 conidia in four separate areas per plate at 200× magnification after incubation at 25 ± 2 °C for 20 h. Four replicate plates were used. Between 90 and 95 % of conidia germinated from the plates used for the experiments.

### Inoculation and mating activity experiments

Flies were contaminated by allowing them to crawl over a 4.7-cm diameter velvet material treated with 0.1 g of dry conidia of *M. anisopliae*. The velvet was inserted inside the top cover (4.8-cm diameter) of a cylindrical plastic tube (9.5 × 4.8 cm), which had the bottom removed and replaced by black nylon netting. Flies were chilled for 2–3 min and transferred to the cylindrical tube. The top cover containing treated velvet was fixed to the tube, and the tube was held topside up (to avoid contact between flies and conidia) until the insects revived. The tube was then turned upside down to allow the flies to walk freely on the velvet. Both sexes of flies were exposed for 10 min to the treatment. In the control treatments, flies were exposed to fungus-free velvet. The velvet was later removed and the insects were kept in the cylindrical plastic tubes for 24 h. Flies were divided into two groups. In the first group, ten 5–7 day old *G. m. morsitans* fungus-treated or fungus-free male flies were transferred individually into polyvinyl chloride (PVC) tsetse-rearing cages (17.0 × 7.0 × 5 cm) and served as “donors.” A fungus-free female fly aged 0–1 day was introduced into each cage and allowed to pair up in copula, and served as the “recipient”. The fungus-free female was isolated immediately after the pair separated and transferred to a clean PVC cage and maintained at room temperature (22–28 °C). An additional 0–1 day-old female was introduced every day for 3 consecutive days (referred to as 1st, 2nd, and 3rd mating line “recipient”) into a clean cage containing either a fungus-contaminated or fungus-free male until three females were used. Flies were transferred individually to clean cages each time the pairs separated as described earlier.

In the second group, 10 each of 0–1 day-old fungus-treated or fungus-free females were transferred individually to PVC cages and served as “donors”. A 5–7 day-old fungus-free male fly was introduced every day up to 3 consecutive days (referred to as 1st, 2nd, and 3rd mating line “recipient”) in each cage containing a fungus-treated or a fungus-free female and was allowed to pair up as above. Flies were maintained at room temperature as described earlier.

In both groups, the time of pairing of each mating couple and the number of jerking movements were noted. The tsetse insemination generally takes place only if the jerking phase is present (Jaenson 1979). Flies were therefore considered as mated when males had jerked. The absence of jerking and thus of mating was simply considered as contact between flies and was also recorded. Mortality was also recorded; dead flies were incubated in 9.0-cm plastic Petri dishes to confirm mycosis. Female flies that survived fungal infection were allowed to larviposite and the number of pupae produced by each fly was recorded up to 60 days following mating.

All experiments were repeated four times.

### Effect of infection by *M. anisopliae* on blood meal intake of *G.m. morsitans* flies

Both male and female *G. m. morsitans* adults were contaminated with conidia of *M. anisopliae* isolate ICIPE 30 using the technique described by Maniania (1994). This technique was used to treat flies with different concentrations of inoculum. The following three concentrations were tested: 3.0 × 10^4^, 3.0 × 10^5^, and 3.0 × 10^6^ conidia ml^−1^. The conidial suspension (10 ml) was placed on a nitrocellulose filter membrane (diameter 47 mm, pore size 0.45 μm, Sigma Chemicals) on an a filter holder unit under vacuum. The filter membrane carrying the conidia was then transferred to the inner side of the lid (diameter 48 mm) of a cylindrical plastic tube (95 × 48 mm) and allowed to air dry for 30 min in a laminar flow cabinet. The cylindrical plastic tube that had the bottom removed and replaced with black plastic netting was used as a chamber for the flies. Flies were chilled for 2–3 min as described earlier and transferred to the tube. The top cover was replaced with the lid containing the treated nitrocellulose membrane and the tube was maintained with the top-side up until the insects revived. The tube was then inverted and the flies were allowed to walk on the membrane for 24 h, after which they were transferred individually into smaller aerated tubes (3 × 5 mm). In the control treatment, sterile distilled water containing 0.05 % Triton X-100 was used. Flies were maintained at a controlled room temperature of 26 ± 2 °C and 70 ± 10 % RH and were offered a blood meal by feeding on a rabbit at 2, 5, 7, 9, and 12 days post-treatment. The blood meal size was calculated by weighing flies before and after the meal. Each treatment consisted of 10 flies and the experiment was replicated four times.

### Data analysis

Data were root-transformed before analysis. A student’s *t* test was used to compare data at *P* = 0.05 significance level. Blood meal weight data were also root-transformed before being subjected to analysis of variance (ANOVA) using PROC GLM, at 95 % level of significance and the Student–Newman–Keuls (SNK) analysis was used to separate the means. A linear regression model was used to study the interactions between dose and sex. All analyses were performed using SAS program version 9.2 (SAS Institute 2008).

## Results

### Transfer of inoculum between flies

Mortality in the fungus-free female and male “recipient” flies (control) that mated or had simple contacts varied between 0 and 20 % (Tables 1, 2). All male flies that were directly exposed to conidia of *M. anisopliae* (i.e., male “donors”) succumbed to fungal infection with mycosis within 10 days post-inoculation. Fungus-free female “recipient” flies that mated with fungus-treated male “donor” flies also died from fungal infection: 87.5, 67.5, and 70 % in the 1st, 2nd, and 3rd line mating, respectively (Table 1). Simple contacts (pairing that did not result in jerking movement) between treated flies and fungus-free flies did not cause high mortality (Table 1). Similarly, all female flies that were directly exposed to conidia of *M. anisopliae* (i.e., female “donors”) succumbed to fungal infection with mycosis occurring within 9 days post-inoculation (Table 2). Mortality in fungus-free male “recipient” flies that mated with fungus-treated female “donor” flies varied between 32.5 and 82.5 % in the 3rd and 1st line mating, respectively (Table 2). Mortality following simple contacts between fungus-treated flies and fungus-free flies varied between 2.5 and 35 % (Table 2).

**Table 1:** Percentage mortality (X ± SE) caused by *Metarhizium anisopliae* in *Glossina morsitans morsitans* female fly “recipients” following successive matings with fungus-treated and fungus-free male “donors”

Treatment	1st line females		2nd line females		3rd line females	
	Mated	Short contact	Mated	Short contact	Mated	Short contact
Fungus-free (control)	15.0 ± 6.5a	0	15.5 ± 2.5a	10.0 ± 4.1a	0a	20.0 ± 5.8a
*M. anisopliae*	87.5 ± 4.5b	0	67.5 ± 4.8b	2.5 ± 2.5b	70.0 ± 4.1a	2.5 ± 2.5b
*t*	55.07	N/A	65.67	62.55	629.65	46.93
*P*	0.0001	N/A	0.003	0.0013	0.0001	0.0082

Four replicates of 10 flies each. Each “donor” fly was allowed to mate three times with different mates at 1-day-interval. Mated copulation: was followed by jerking, Short contact: attempts by male flies to copulate with females. Means (±SE) within column followed by the same letter are not significantly different by *t* test (*P* > 0.05)

**Table 2:** Percentage mortality (X ± SE) caused by *Metarhizium anisopliae* in *Glossina morsitans morsitans* male fly “recipients” following successive matings with fungus-treated and fungus-free female “donors”

Treatment	1st line males		2nd line males		3rd line males	
	Mated	Short contact	Mated	Short contact	Mated	Short contact
Fungus-free (control)	12.5 ± 6.3a	0a	7.5 ± 2.5 a	10.0 ± 4.1a	0a	20.0 ± 5.8a
*M. anisopliae*	82.5 ± 2.5b	2.5 ± 2.5b	37.5 ± 16.5b	35.0 ± 13.2b	32.5 ± 11.8b	27.5 ± 11.1a
*t*	64.61	68.81	45.53	52.63	366.91	15.59
*P*	0.0031	0.0028	0.001	0.0014	0.0001	0.0239

Four replicates of 10 flies each. Each “donor” fly was allowed to mate three times with different mates at 1-day-interval. Mated copulation: was followed by jerking, Short contact: attempts by male flies to copulate with females. Means (±SE) within columns followed by the same letter are not significantly different by *t* test (*P* > 0.05)

### Mating activity of *G.m. morsitans* males

**Fig. 1:**
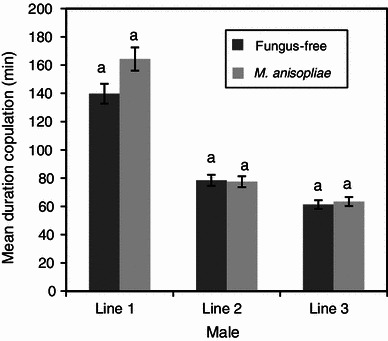
Mean duration of copulation (min) of fungus-free male “recipient” *Glossina morsitans morsitans* mated with *Metarhizium anisopliae*-treated and fungus-free female “donors”. Four replicates of 10 flies each. Each “donor” fly was allowed to mate three times with different mates at 1-day-interval. *Bars* denote means ± one standard error (*P* = 0.05, *t* test)

**Fig. 2:**
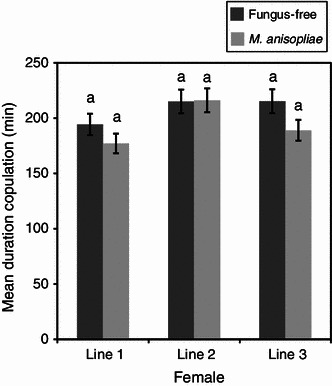
Mean duration of copulation (min) of *Metarhizium anisopliae*-treated and fungus-free male “donor” *Glossina morsitans morsitans* mated with fungus-free female “recipients”. Four replicates of 10 flies each. Each “donor” fly was allowed to mate three times with different mates at 1-day-interval. *Bars* denote means ± one standard error (*P* = 0.05, *t* test)

**Fig. 3:**
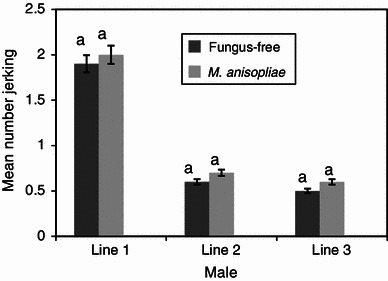
Mean number of jerking movements of fungus-free male “recipient” *Glossina morsitans morsitans* mated with *Metarhizium anisopliae*-treated and fungus-free female “donors”. Four replicates of 10 flies each. Each “donor” fly was allowed to mate three times with different mates at 1-day-interval. *Bars* denote means ± one standard error (*P* = 0.05, *t* test)

**Fig. 4:**
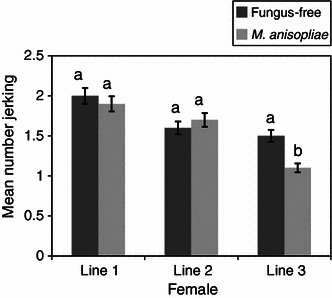
Mean number of jerking movements of fungus-free female “recipient” *Glossina morsitans morsitans* mated with *Metarhizium anisopliae*-treated and fungus-free male “donors”. Four replicates of 10 flies each. Each “donor” fly was allowed to mate three times with different mates at 1-day-interval. *Bars* denote means ± one standard error (*P* = 0.05, *t* test)

Both the fungus-treated and the fungus-free males (control) were equally efficient in mating. They located the females and manifested a very high level of sexual activity. The mean duration of copulation of fungus-treated and fungus-free males was not significantly different (*P* > 0.05) for all the mating lines (Figs. 1, 2). The duration of copulation was 140 and 164 min in fungus-free and fungus treatments, respectively, when female flies served as “donors” in the 1st line mating and declined in subsequent line matings, 61.3 and 63.4 min in fungus-free and fungus-treated treatments, respectively, in the 3rd line mating (Fig. 1). When male flies were used as “donors”, the mean duration of copulation remained similar for both the fungus and fungus-free treatments in all the three line matings (Fig. 2). The total number of jerking movements between the fungus-free and the fungus-treated flies was also not significantly different (*P* > 0.05), except when male flies mated with the 3rd line female flies (*P* *<* 0.01) (Figs. 3, 4). The number of jerking movements was 1.9 and 2.0 in fungus-free and fungus treatments, respectively, in the 1st line mating and reduced in subsequent mating lines (0.5 and 0.6 in fungus-free and fungus treatments, respectively, in the 3rd line mating), when female flies served as “donors” (Fig. 3). When male flies served as “donors”, the number of jerking movements was 2.0 and 1.9 in fungus-free and fungus treatments, respectively, in the 1st line mating and dropped to 1.5 and 1.1 in fungus-free and fungus treatments, respectively, in the 3rd line mating (Fig. 4).

### Receptivity of fungus-treated or free-fungus female flies with fungus-treated or fungus-free males

In the 1st line mating, all the female flies in the different groups displayed a very high level of sexual receptivity. For all treatments, as soon as a male was introduced into the cage with a female, it grabbed the female, who immediately opened her wings and mating started. Receptivity dropped significantly in the subsequent matings as illustrated by the duration of copulation and the number of jerkings. However, there were no significant differences between the treatments (Figs. 1, 2, 3, 4).

### Effect of infection by *M. anisoplaie* on fertility of *G.m. morsitans* females

Infection by *M. anisopliae* significantly affected the fertility of female flies that survived infection in the 1st (*t* = 6.93; *P* = 0.001) and 2nd (*t* = 5.91; *P* = 0.005) line mating, with fungus-free females being more fertile than fungus-treated females (Table 3). Females that survived fungal infection between 17 and 35 days (the first larviposition generally occurs after 17 days) produced fewer pupae in the 1st (*t* = 2.83; *P* < 0.03) and 2nd (*t* = 3.0; *P* = 0.02) line in fungus than in fungus-free treatments (Table 3). Over the 60-day experimental period, fungus-free females produced more pupae per female than did the fungus-treated females, but the difference was only significant for the 1st line (*t* = 4.2; *P* < 0.01) and 3rd line mating (*t* = 437; *P* = 0.01) (Table 3).

**Table 3:** Effect of infection by *Metarhizium anisopliae* on reproduction potential of *Glossina morsitans morsitans* females

Treatment	1st line females	2nd line females	3rd line females
*No. fertile females (X* *±* *SE)*			
Fungus-free (control)	7.5 ± 0.6a	7.5 ± 0.6a	6.0 ± 0.9a
*M. anisopliae*	3.4 ± 0.5b	3.5 ± 0.9b	6.0 ± 1.1a
*t*	6.92	5.90	N/A
*P*	0.001	0.005	N/A
*No. pupae/female (X* *±* *SE) per female produced between 1 and 35* *days after infection* ^*1*^			
Fungus-free (control)	2.3 ± 0.2a	2.5 ± 0.2a	2.0 ± 0.1a
*M. anisopliae*	1.2 ± 0.1b	1.4 ± 0.2b	1.4 ± 0.3a
*t*	2.82	3.0	1.32
*P*	0.030	0.025	0.245
*No. pupae (X* *±* *SE)* ^*2*^			
Fungus-free (control)	4.0 ± 0.5a	3.5 ± 0.5a	3.5 ± 0.6a
*M. anisopliae*	1.4 ± 0.3b	2.5 ± 0.9a	2.1 ± 0.4b
*t*	4.2	1.41	4.37
*P*	0.013	0.227	0.011

Four replicates of 10 flies each. Female flies mated with *Metarhizium anisopliae*-treated and fungus-free male ‘donor’

^1^Number of pupae/fly produced by fungus-treated flies that survived up to 35 days post-infection, and free-fungus flies for the same period

^2^Number of pupae produced per female over a period of 60 days post-infection. Means within-column followed by the same letter are not significantly different by *t* test (*P* > 0.05)

### Effect of infection by *M. anisopliae* on blood feeding

Since most flies had died by day 9 post-treatment at the higher concentration, data of 7 days post-treatment were analyzed. The weight of blood meal intake by female flies was generally higher than the one by the male flies. In the control for example, the blood meal weight taken by single female fly was 121.3 mg compared to 96.8 mg by male fly at 7 day post-treatment. The effect of fungal infection on blood meal intake was significantly different (*P* < 0.01) only in insects treated with the high concentration of 3.0 × 10^6^ conidia ml^−1^ and was observed in both males and females (Table 4). Interaction was significant (*P* = 0.0001) with dose and sex but not with Dose × Sex (*P* = 0.5232) (Table 4).

**Table 4:** Weight (mg) of blood meal intake by *Glossina morsitans morsitans* following infection by *Metarhizium anisopliae*

Treatment	Blood weight (mg) (X ± SE)	
	Sex	
	Female	Male
Control	121.3 ± 5.8a	96.8 ± 5.8a
3 × 10^4^ conidia ml^−1^	118.5 ± 5.9a	84.3 ± 3.0a
3 × 10^5^ conidia ml^−1^	110.5 ± 8.9a	81.3 ± 9.2a
3 × 10^6^ conidia ml^−1^	79.0 ± 6.8b	51.3 ± 6.5b
	*F* = 5.06, df = 3.9, *P* = 0.0155	*F* = 5.58, df = 3.9, *P* = 0.0113
Interaction	Dose (*F* = 17.74, df = *P* = < 0.0001), Sex (*F* = 50.04, *P* = < 0.0001), Dose × Sex (*F* = 0.83, *P* = 0.5232)	

Means within-column followed by the same letter are not significantly different by ANOVA (*P* > 0.05)

## Discussion

In the present study, the mating behavior of *M. anisopliae*-infected *G.m. morsitans* flies was similar to that of fungus-free (control) flies under laboratory conditions since no difference in the number of jerking movements and duration of mating was observed between the two treatments. Similar observations have been reported for other Diptera. Toledo et al. (2007) reported that mating success of the Mexican fruit fly, *Anastrepha ludens* (Loew), was not affected by fungal infection over 3 days post-inoculation. Similar observations were reported on three African tephritids, *Ceratitis* *capitata* (Wiedemann)*, C. cosyra* (Bezzi), and *C. fasciventris* (Walker) (Dimbi et al. 2009). Successful mating (copulation followed by jerking movement) could therefore explain why a single fungus-contaminated fly could transfer inoculum to as many as three healthy mates and infect them. Horizontal transmission of inoculum has already been reported by Meadow et al. (2000) with *Beauveria bassiana* (Bals.) Vuill. against the cabbage maggot, *Delia radicum* L., where transfer of inoculum was possible for a series of at least six flies, Toledo et al. (2007) with *B. bassiania* on *A. ludens*, and Quesada-Moraga et al. (2008) with *M. anisopliae* on *C.* *capitata*. In our study, mycosis by *M. anisopliae* was greater in the 1st line than in the subsequent line matings, suggesting a decline in the amount of conidia transferred/acquired. Turner (1980) noted that the amount of fluorescent powder transferred from marked males to female *G. m. morsitans* declined with the number of matings in the laboratory. However, the decline in mortality was less pronounced in female “recipient” flies (Tables 1, 2). The difference in susceptibility of the sexes to fungal infection could explain this. For instance, Maniania and Odulaja (1998) reported that female *G.m. morsitans* were more susceptible to *M. anisopliae* than males. The authors hypothesized that the morphology of female genitalia which is composed of sclerotized plates surrounding the anus and valves allows firm adherence of conidia as opposed to male genitalia which are characterized by a button-like hypopygium. On the other hand, it has been reported in *C. capitata* that males were good vectors in terms of transmission of fungal inoculum compared to females (Quesada-Moraga et al. 2008). In both cases, refractoriness of female flies to multiple matings should also be taken into account. A fungus formulation that is electrostatically charged or lipophilically active may therefore enhance the adherence of conidia on the cuticle of flies and increase the transmission of inoculum.

Tsetse female receptivity decreased after the 1st mating. Pre-mated tsetse females generally rebuffed subsequent males attempting to mate. But even in such attempts, males mounted females, which desisted from copulating after a few moments. Despite brief attempts at copulation, male flies were able to contract infection from *M. anisopliae*-treated females (Table 2). Toledo et al. (2007) reported similar observations in *A. ludens.* Mating was successful (100 %) in the 1st line mating in both fungus-free and *M. anisopliae*-treated females; but females acquired sexual refractoriness thereafter (Table 2). The number of fertile female flies surviving infection was significantly different between fungus-free and fungus-treated insects in the 1st and 2nd line mating (Table 3), which is in concordance with the observations of Toledo et al. (2007) who reported that fungal infection notably reduced fertility of *A. ludens* female flies.

The infection by *M. anisopliae* on blood meal intake had an effect only at the higher concentration. In mosquitoes, reduction in blood meal intake following infection by entomopathogenic fungi has been associated with reduction in transmission potential of the parasite (Blanford et al. 2005; Scholte et al. 2006). In general, female flies that mated with *M. anisopliae*-treated males produced fewer pupae than fungus-free flies, although the difference was only significant in few cases. According to Boyle (1971), half of the blood ingested by tsetse such as *Glossina austeni* Newstead serves in pupae production. The reduction in number of pupae laid by fungus-infected females could be therefore attributed to the reduced amount of blood meal taken by those infected flies.

In conclusion, this study has shown that fungal infection does not affect the mating behavior of tsetse flies in the laboratory, and fly-to-fly contamination does occur during matings. These are important attributes if entomopathogenic fungi have to be used in auto-dissemination strategy and be integrated into sterile insect technique (SIT) as currently being advocated (Maniania and Ekesi 2012).

## References

[CR1] Blanford S, Chan BH, Jenkins N, Sim D, Turner RJ, Read AF, Thomas MB (2005). Fungal pathogen reduces potential for malaria transmission. Science.

[CR2] Boyle JA (1971). Effect of blood intake of *Glossina austeni* Newst. on pupal weights in successive reproductive cycles. Bull Entomol Res.

[CR3] Castillo MA, Moya P, Primo-Yúfera E (2000). Susceptibility of *Ceratitis capitata* Wiedemann (Diptera: Tephritidae) to entomopathogenic fungi and their extracts. Biol Control.

[CR25] Dimbi S, Maniania NK, Ekesi S (2009) Effect of *Metarhizium anisopliae* inoculation on the mating behavior of three species of African Tephritid fruit flies, *Ceratitis capitata*, *Ceratitis cosyra* and *Ceratitis fasciventris*. Biol Control 50:111–116

[CR4] Eilenberg J (1987). Abnormal egg laying behavior of female carrot flies (Psila rosae) induced by fungus *Entomophthora muscae*. Entomol Exp Appl.

[CR5] Goettel MS, Inglis DG, Lacey LA (1997). Fungi hyphomycetes. Manual of techniques in insect pathology.

[CR6] Howard AFV, N’Guessan R, Koenraadt CJM, Asidi A, Farenhorst M, Akogbéto M, Thomas MB, Knols BGJ, Takken W (2010). The entomopathogenic fungus *Beauveria bassiana* reduces instantaneous blood feeding in wild multi-insecticide-resistant *Culex quinquefasciatus* mosquitoes in Benin, West Africa. Parasite Vector.

[CR7] Jaenson TGT (1979). Mating behaviour of males of *Glossina pallidipes* Austen (Diptera: Glossinidae). Bull Entomol Res.

[CR8] Kaaya GP, Okech MA (1990). Horizontal transmission of mycotic infection in adult tsetse, *Glossina morsitans morsitans*. Entomophaga.

[CR9] Maniania NK (1994). A laboratory technique for infecting adult tsetse with a fungal pathogen. Insect Sci Appl.

[CR10] Maniania NK (2002). A low-cost contamination device for infecting adult tsetse flies, *Glossina* spp., with the entomopathogenic fungus *Metarhizium anisopliae* in the field. Biocontrol Sci Technol.

[CR11] Maniania NK, Ekesi S (2012) Use of entomopathogenic fungi in the control of tsetse flies. J Invertebr Pathol: 10.1016/j.jip.2012.07.019 22841947

[CR12] Maniania NK, Odulaja A (1998). Effect of species, age and sex of tsetse on response to infection by *Metarhizium anisopliae*. Biocontrol.

[CR13] Maniania NK, Ekesi S, Odulaja A, Okech MA, Nadel DJ (2006). Prospects of a fungus-contamination device for the control of tsetse fly *Glossina fuscipes fuscipes*. Biocontrol Sci Technol.

[CR14] Meadow R, Vandenberg JD, Shelton M (2000). Exchange of inoculum of *Beauveria bassiana* (Bals.) Vuill. (Hyphomycetes) between adult flies of the cabbage maggot *Delia radicum* L. (Diptera: Anthomyiidae). Biocontrol Sci Technol.

[CR15] Mnyone LL, Kirby MJ, Mpingwa MW, Lwetoijera DW, Knols BGJ, Takken W, Koenraadt CJM, Russell TL (2011). Infection of *Anopheles gambiae* mosquitoes with entomopathogenic fungi: effect of host age and blood-feeding status. Paras Res.

[CR16] Ondiaka S, Bukhari T, Farenhorst M, Takken W, Knols BGJ (2008). Effects of fungal infection on the host-seeking behaviour and fecundity of the malaria mosquito *Anopheles gambiae* Giles. Proc Neth Entomol Soc Meet.

[CR17] Quesada-Moraga E, Martin-Carballo I, Garrido-Jurado I, Santiago-Álvarez C (2008). Horizontal transmission of *Metarhizium anisopliae* among laboratory populations of *Ceratitis capitata* (Wiedemann) (Diptera: Tephritidae). Biol Control.

[CR18] SAS Institute (2008) SAS/STAT^®^, release 9.2 User’s Guide. Cary

[CR19] Schaechter E (2000). Weird and wonderful fungi. Microbiol Today.

[CR20] Scholte EJ, Knols BGL, Takken W (2004). Autodissemination of the entomopathogenic fungus *Metarhizium anisopliae* amongst adult of the malaria vector *Anopheles gambiae*. Malaria J.

[CR21] Scholte E-J, Knols BGJ, Takken W (2006). Infection of the malaria mosquito *Anopheles gambiae* with the entomopathogenic fungus *Metarhizium anisopliae* reduces blood feeding and fecundity. J Invertebr Pathol.

[CR22] Toledo J, Campos SE, Flores S, Liedo PF, Barrera J, Villaseñor A, Montoya P (2007). Horizontal transmission of *Beauveria bassiana* in *Anastrepha ludens* (Diptera: Tephritidae) under laboratory and field cage conditions. J Econ Entomol.

[CR23] Turner DA (1980). A novel marking/release/recapture method for possible use in determining aspects of tsetse fly behaviour. Insect Sci Appl.

[CR24] Watson DW, Petersen JJ (1993). Sexual activity of Male *Muscae domestica* (Diptera: Muscidae) infected with *Entomophthora muscae* (Entomophthoraceae: Entomophthorales). Biol Control.

